# Adherence to Integrated Management of Childhood Illnesses Guideline in Treating South Sudanese Children with Cough or Difficulty in Breathing

**DOI:** 10.1155/2017/5173416

**Published:** 2017-09-18

**Authors:** Jonathan Izudi, Stanley Anyigu, David Ndungutse

**Affiliations:** ^1^Institute of Public Health and Management, International Health Sciences University, P.O. Box 7782, Kampala, Uganda; ^2^Uganda Society for Health Scientists, Makerere University College of Health Sciences, Department of Anatomy, P.O. Box 7072, Kampala, Uganda; ^3^The International Rescue Committee, South Sudan Program, Juba, South Sudan; ^4^School of Public Health, St. Augustine International University, P.O. Box 88, Kampala, Uganda

## Abstract

**Background:**

Pneumonia substantially kills children aged 2–59 months in South Sudan. However, information on health workers adherence to Integrated Management of Childhood Illnesses (IMCI) guideline in treating children with cough/difficulty in breathing remains scarce. This study assessed factors associated with adherence to IMCI guideline in Aweil East County, South Sudan.

**Methods:**

This cross-sectional study involved 232 health workers from 36 health facilities. Data collected using structured questionnaire and checklist was double-entered in EpiData and analyzed with STATA at 5% significance level using logistic regression.

**Results:**

Respondents mean age was 32.41 ± 7.0 years, 154 (66.4%) were males, 104 (44.8%) reached secondary education, and 190 (81.9%) had certificate. 23 (9.9%, 95% CI: 6.4–14.5) adhered to IMCI guideline. Holding diploma (adjusted odds ratio (AOR) = 6.97; 95% Confidence Interval (CI): 1.82–26.67; *P* = 0.005), shorter time to follow guideline steps (AOR = 12.0; 95% CI: 2.73–61.66; *P* < 0.001), and nondifficult use (AOR = 27.7; 95% CI: 5.40–142.25; *P* < 0.001) were associated with adherence.

**Conclusion:**

Adherence was low. Academic qualifications, guideline complexity, and availability of IMCI drugs were associated factors.

## 1. Background

Globally, pneumonia is recognized as a leading cause of under-five mortality. Pneumonia alone is responsible for more deaths than combined deaths from malaria, tuberculosis, and Human Immunodeficiency Virus (HIV) in this category of children. South Asia and Sub-Saharan Africa carry the heaviest global burden of pneumonia estimated at 18% and 90% deaths among children under-five years, respectively [1–3]. To address this problem, the World Health Organization (WHO) formulated guideline of integrated management of child illnesses (IMCI) to direct health workers in assessing and managing pneumonia step by step in children presenting with cough and difficulty in breathing (DIB) at health facilities [4]. Pneumonia is a leading cause of under-five mortality in South Sudan, accounting for 19% of all childhood deaths [5]. Consequently, the IMCI guideline was introduced by Government of South Sudan in 2007 to address the high mortality rates in selected health facilities due to shortage of Human Resources for Health [6].

In Aweil East County (AEC), Northern Bar El Ghazal State (NBEGS), South Sudan, reports indicate pneumonia is incorrectly diagnosed and treated in spite of the presence of the IMCI guideline [7]. Without addressing this practice, it is obvious that most, if not all, children with pneumonia will be incorrectly managed resulting in antibiotic resistance and high mortality rates [3]. In this study, we assessed the level and factors associated with health worker adherence to the IMCI guideline when treating children with cough or DIB in AEC, NBEGS, South Sudan.

## 2. Methods

This study was conducted at 36 health facilities in AEC, NBEGS, South Sudan. Health services delivery in South Sudan is provided at four different levels (central, state, county, and community levels) each with a different diagnostic capacity and staffing requirements. Health services are further categorized as community, primary health, secondary, and specialized care interlinked with a referral system [8]. Community health workers, maternal and child health workers, and Home Health Promoters provide community healthcare at Primary Healthcare Units (PHCUs) and Primary Healthcare Centers (PHCCs) as entry points. PHCUs provide the first level of interaction between the community and the formal health system in providing basic preventive, promotive, and curative care to about 15,000 people. PHCCs provide diagnostic/laboratory services and maternity care to an estimated 50,000 people alongside services provided at PHCUs. County and State Hospital levels provide secondary, comprehensive-obstetric, inpatient, and surgical care to an estimated 300,000 and 500,000 people, respectively [8].

This study used an analytical cross-sectional design to assess and determine health workers' adherence to the IMCI guideline when treating children under 5 years with cough or DIB. The 36 health facilities (one State Hospital, one County Hospital, six PHCCs, and 28 PHCUs) were purposively selected but respondents were consecutively selected health workers that treated children aged 2–59 months with cough or DIB at the outpatient and pediatric units.

Within 95% confidence limits, 5% precision, 50% estimated adherence to the IMCI guideline, and 360 health workers previously trained in the IMCI guideline in AEC, 232 health workers based on Krejcie and Morgan (1970) formula [9, 10] were required in this study.

Data was collected by trained and supervised Research Assistants between November 2016 and January 2017 using a standardized pretested questionnaire and observation checklist adopted from the WHO IMCI flowchart for managing children with cough or DIB. Nine questions on assessing, documenting presenting complaints, performing physical inspection, and prescribing appropriate treatment to measure health worker adherence to the IMCI guideline were developed from the WHO IMCI flowchart:

1. Asking about the child's ability to drink or breast-feed
2. Asking about the child's current or past vomiting history
3. Asking for history of convulsions
4. Checking the child for lethargy or unconsciousness
5. Asking about cough and its duration
6. Assessing the child's breathing patterns by taking off the child's clothes and looking at the chest movements
7. Assessing the child's respiratory rate by counting the number of breaths/minute
8. Correctly classifying the cough or DIB
9. Prescribed antibiotics as per the IMCI guideline.

The Research Assistants were health workers with at least 5 years of experience in the IMCI clinical practice.

The outcome variable was adherence to the IMCI guideline when treating children aged 2–59 months with cough or DIB measured on a dichotomous scale (“no” or yes). To be adherent, a health worker had to correctly follow all the nine steps in the IMCI flowchart. Failure to adhere to any one of the nine steps constituted nonadherence. The independent variables were health facility staffing levels, availability of the IMCI guideline, presence of essential IMCI drugs and supplies, staff workload, frequency of IMCI support supervisions, importance attached to the IMCI guideline by a health workers, perceived work load, previous training in the IMCI guideline, attitudes of health workers towards the IMCI guideline, perceived benefits of the IMCI guideline on patient waiting time, cadre and qualifications of health workers, years of service, and perceived motivation in using the IMCI guideline.

Data was double entered in EpiData version 3.1 (EpiData Association, Odense, Denmark) [11] and exported to STATA version 12 (StataCorp, College Station, TX, USA) for univariate, bivariate, and multivariate analysis. Univariate analysis involved the computation of descriptive statistics of means and standard deviations (SD) for numerical variables and medians with interquartile ranges (IQR) for categorical variables. In bivariate analysis, the association between categorical independent variables and adherence to IMCI guideline was examined with the Chi-squared test for larger cell counts or Fisher's exact for smaller cell counts, and Student's *t*-test was used when the independent variable was numerical. Test of collinearity between variables was performed and variables with variance inflation factor greater than ten (VIF > 10) were excluded from the final model.

However, none of the variables were collinear. In the final stepwise backward multivariate logistic regression model, significant variables at bivariate analysis and IMCI technical support supervision as a clinically relevant variable were included and the output was stated in odds ratios, 95% Confidence Intervals, and *P* values. Level of statistical significance was set at 5% all through.

This study was approved by the Institutional Review Board of International Health Sciences University and AEC Health Department. In addition, approval was obtained from respective Health Facility Heads. Study participants likewise gave informed written consent after explaining the purpose, benefits, and risks involved. They were free to withdraw their participation in the study at any time.

## 3. Results

### 3.1. Sociodemographic Characteristics of Participants

The mean age of the respondents was 32.41 ± 7.0 years (median, 32 years; IQR: 28–37), 154 (66.4%) were males, 71 (30.6%) were Anglicans, 104 (44.8%) ended their education at secondary level, 190 (82.0%) had certificates in medical training, and 124 (53.5%) were community health workers (CHWs) (Table 1).

### 3.2. Adherence to the IMCI Guideline

Of 232 participants, 193 (83.2%) had asked the mother about the child's ability to drink or breast-feed, 137 (59.1%) had asked about vomiting/vomited everything, 139 (59.9%) had asked about convulsions, 122 (52.6%) had asked about cough and its duration, 153 (66.0%) assessed or looked for lethargy or level of consciousness, 144 (62.1%) took off the child's clothes and observed for abnormal breathing patterns, 28 (18.7%) counted and recorded the child's respiratory rate, 151 (65.1%) classified the presenting complaint correctly, and 186 (80.2%) gave the correct antibiotics. Overall, 23 (9.9%: 95% CI: 6.4–14.5) respondents adhered to all the steps of the IMCI guideline when treating children with cough or DIB (Table 2).

### 3.3. Bivariate Analysis of Factors Associated with Adherence to IMCI Guideline

Table 3 shows 18 (14.0%) respondents aged 32 years or less compared to five (4.8%) above 32 years that were adherent (*P* = 0.02). Eight (4.2%) respondents that had certificates, 13 (35.1%) that had diploma, and two (40.0%) that had bachelor's degree were adherent (*P* < 0.001).

Two (2.0%) respondents that stated it took a long time to follow the steps of the IMCI guideline in contrast to 21 (15.7%) that reported it took a short time were adherent (*P* = 0.009). 14 (7.8%) respondents that had never received technical support supervision in the IMCI guideline compared to 9 (17.0%) that had ever received were adherent (*P* = 0.05). Four (3.8%) respondents that reported the number of staffs in the health facility was inadequate to treat children compared to 19 (15.1%) that stated it was adequate were adherent (*P* = 0.004). 11 (6.5%) respondents that reported recent shortage of IMCI drugs in the preceding month compared to 12 (19.0%) that reported no shortage were adherent (*P* = 0.004). Two (2.0%) respondents that had difficulty in using the IMCI guideline and 21 (16.1%) that had no difficulty in using the guideline were adherent (*P* < 0.001).

### 3.4. Univariable Analysis of Factors Associated with Adherence to IMCI Guideline

In unadjusted analysis (Table 3), respondents aged more than 32 years compared to less than or equal to 32 years had reduced adherence (unadjusted odds ratio (UOR) = 0.31; 95% CI: 0.11–0.88, *P* = 0.027). Conversely, adherence increased with holding a diploma (UOR = 12.32; 95% CI: 4.63–32.77, *P* < 0.001) or degree (UOR = 15.17; 95% CI: 2.21–103.89, *P* = 0.006), short time taken to follow steps of the guideline (UOR = 8.92; 95% CI: 2.04–39.02; *P* = 0.004), ever receiving IMCI technical support supervision (UOR = 2.41; 95% CI: 0.98–5.94, *P* = 0.056), adequate staffing at health facility (UOR = 4.53; 95% CI: 1.49–13.76; *P* = 0.008), no recent shortage of IMCI drugs (UOR = 3.38; 95% CI: 1.41–8.12; *P* = 0.006), and no difficulties in using the guideline (UOR = 9.63; 95% CI: 2.20–42.13; *P* = 0.003).

### 3.5. Multivariate Analysis of Factors Associated with Adherence to IMCI Guideline

After adjusting for health worker academic qualifications, age, IMCI support supervision, staffing, time taken to follow the steps of the guideline, difficulties in using the guideline and availability of IMCI drugs (Table 3), holding a diploma (AOR = 6.97; 95% CI: 1.82–26.67; *P* = 0.005) compared to certificate, a short time taken to follow the steps of the IMCI guideline (AOR = 12.0; 95% CI: 2.73–61.66; *P* < 0.001), no recent shortage of IMCI drugs (AOR = 7.11; 95% CI: 2.37–21.38; *P* < 0.001), and absence of difficulties in using the guideline (AOR = 27.7; 95% CI: 5.40–142.25; *P* < 0.001) were associated with increased adherence. However, maternal age above 32 years (AOR = 0.63; 95% CI: 0.19–2.15; *P* = 0.463), holding a degree (AOR = 1.02; 95% CI: 0.10–10.43; *P* = 0.986), ever receiving the IMCI technical support supervision (AOR = 0.94; 95% CI: 0.27–3.24; *P* = 0.921), and adequate staffing at the health facility (AOR = 2.96; 95% CI: 0.85–10.32; *P* = 0.089) were not associated with adherence.

## 4. Discussion

This study assessed the level and factors associated with health worker adherence to the IMCI guideline when treating children with cough or DIB in AEC, NBEGS, South Sudan. The level of adherence was low at 9.9% suggesting most children that presented at the health facilities with cough or DIB were inappropriately managed. In Tanzania [12] and elsewhere [13], low adherence to the IMCI guideline was previously reported [12, 13]. According to the WHO, at least two in every five health workers must adhere to the IMCI guideline for effective management of childhood pneumonia [14]. With low health worker adherence to the IMCI guideline, the potential impact of the IMCI strategy on under-five morbidity and mortality is thus lost [15]. Our results compare unfavorably with those in Benin and Senegal where 64% and 16% of health workers, respectively, were adherent [16].

In this study, the major steps in the WHO IMCI flowchart commonly missed by health workers include asking for vomiting, convulsions, cough or DIB, and the duration, assessing for lethargy or level of consciousness and counting the respiratory rate. Similar gaps in following the steps of the IMCI guideline were observed in Benin, Senegal, Tanzania, and Botswana [12, 16, 17]. However, our findings reflect a much lower level of adherence compared to the studies in Benin, Senegal, and Botswana. These gaps should be addressed through onsite mentorships of health workers in using the IMCI guideline followed by technical support supervision. Earlier in Tanzania, incorrect antibiotics were prescribed to children diagnosed with severe pneumonia [12].

Similarly in Botswana, children were prescribed incorrect antibiotics after diagnosis of pneumonia [18]. Our result is therefore not surprising because it generally appears that there is lack of adherence to the IMCI guideline among health workers in Sub-Saharan Africa.

This study found diploma and degree compared to certificate holders were more adherent to the IMCI guideline. In South Sudan, the health worker force is dominated by CHWs usually trained for 9 months. Their medical skills and knowledge are therefore unmatched with diploma and degree holders usually trained for minimally 3 years. Our results contradict with previous study in Tanzania that found low adherence to IMCI guidelines among clinicians with better knowledge of IMCI [13]. Additionally, it controverts earlier evidence that well-trained health workers in rural and hard to reach areas hardly stick to guidelines set for treating pneumonia [19]. Regardless of previous evidences, level of training and academic qualifications might determine adherence to national and international guidelines for disease management. A preservice training in the IMCI guideline might thus be critical in the training of CHWs so as to increase adherence [20] particularly in AEC, South Sudan.

We found adherence to the IMCI guideline increased with no reported recent shortage of IMCI drugs. This was not surprising because availability of IMCI drugs ensures correct prescription as per defined standards. Our result agrees with a study in Kenya [21] that found high adherence to the IMCI guideline at health facilities without shortage of IMCI drugs.

In this study, respondents that indicated it takes a short time to follow the steps of the IMCI guideline and that it is not difficult to use the guideline had increased adherence. This result implies that the IMCI guideline is less adhered to by health workers that face difficulty in following its steps resulting in lengthy time requirement. The combination of these two findings suggests that guideline complexity or simplicity is a crucial determinant of adherence. Our result is in conformity with an earlier study that evaluated the performance of CHWs in managing multiple childhood illness at outpatient and inpatient units in Siaya district of Kenya, where mistakes occurred in symptom assessment, disease classification, and prescription of correct doses of medications due to guideline complexity and inadequate supervision [22]. In addition, it is consistent with results of a past study in Senegal that found low adherence to the IMCI guideline on regular basis among Medical Doctors, Nurses, and Midwives after IMCI training due to difficulties encountered in following the required steps [16].

Another supporting evidence of our findings comes from previous study that assessed the safety and effectiveness of modifying the IMCI guideline that initially resulted in most children with severe pneumonia being treated locally at first-level facilities. In the study, the modification of the IMCI guideline increases the number of children that were correctly managed and reduced unnecessary referrals [23]. These research evidences [16, 22–24] confirm that difficulties in using the IMCI guideline are a common phenomenon that probably accounts for low adherence. In enhancing adherence to the IMCI guideline, capacity building of health workers through onsite mentorships, in-service training, and Continuous Professional Education (CPE) is important.

In Kenya, CPE sessions and clinical mentorships increased health worker adherence to the IMCI guideline [21]. However in South Sudan, CPE remains a critical challenge of HRH [25], a factor that would have improved HRH capacity in adhering to national and international guidelines.

## 5. Study Limitations

This study established low adherence to the IMCI guideline in treating children below 5 years with cough or DIB in AEC, NBEGS, South Sudan. Nevertheless, it has a number of drawbacks. First, there is possibility of interobserver and intraobserver variation when observing health workers in following the steps of the IMCI guideline. However, we minimized this pitfall by using health workers that had experience in the IMCI as Research Assistants. Secondly, self-reporting bias is likely especially in providing information on ever receiving technical support supervision, reporting shortage of IMCI drugs, and difficulties in following the steps of the IMCI guideline. The absence of data on the actual number of health workers in the units so as to establish the staffing norm is another limitation that must be considered in the interpretation of our results.

## 6. Conclusion and Recommendations

Our study is the first in AEC, NBEGS, South Sudan, to indicate low adherence of health workers to the IMCI guideline when treating children under five years of age with cough or DIB. Adherence was high among well-trained health workers that held diploma or degrees, among health workers that took a short time to follow the guideline steps, in health facilities where no shortage of IMCI drugs was reported, and when health workers had no difficulty in using the guideline. In improving health worker adherence to the IMCI guideline in South Sudan, the Government should address shortages of HRH (Human Resources for Health) by recruiting and retaining well-trained high-level professionals, building the capacity of existing health workforce in the IMCI guideline and ensuring a sustainable supply of IMCI drugs.

## Figures and Tables

**Table 1:** Characteristics of respondents.

Variable	Frequency (number = 232)	Percentage (% = 100)
*Age (years)*		
19–32	129	55.6
33–55	103	44.4
Age in years (mean ± SD)	232	32.41 ± 7.0
*Sex*		
Female	78	48.3
Male	154	66.4
*Religion*		
Catholic	112	48.3
Moslem	43	18.5
Anglican	71	30.6
Others	6	2.6
*Highest level of education*		
Primary	58	25.0
Secondary	104	44.8
Tertiary	70	30.2
*Academic qualifications*		
Certificate	190	81.9
Diploma	37	16.0
Undergraduate	5	2.2
*Designation*		
Community health worker	124	53.5
Nursing officer	69	29.7
Clinical officer	34	14.7
Medical officer	5	2.2

**Table 2:** Adherence to IMCI guideline in the treatment of children with cough or difficulty in breathing in Aweil East County, South Sudan.

Variable	Number = 232	Percentage (% = 100)
Health worker asked the mother whether the child was able to drink or breast-feed		
Yes	193	83.2
No	39	16.8
Health worker asked the mother whether the child was vomiting/vomited everything		
Yes	137	59.1
No	95	40.9
Health worker asked the mother whether the child had had convulsions		
Yes	139	59.9
No	93	40.1
Health worker asked the mother if the child had had cough and for how long		
Yes	122	52.6
No	110	47.4
Health worker assessed or looked at the child for lethargy or level of consciousness		
Yes	153	66.0
No	79	44.0
Health worker took off the cloth of the child and looked at the chest		
Yes	144	62.1
No	88	37.9
Health worker counted the respiratory rate of the child		
Yes	28	18.7
No	122	81.3
Health worker classified the pneumonia correctly		
Yes	151	65.1
No	81	34.9
Health worker gave the correct antibiotics as per the IMCI guideline		
Yes	186	80.2
No	46	19.8
Adherence to the IMCI guideline		
No	23	9.9
Yes	209	90.1

**Table 3:** Unadjusted and adjusted analysis of factors associated with adherence to the IMCI guideline when treating children with cough or difficulty in breathing in Aweil East County, Northern Bahr El Ghazal State, South Sudan.

Variable	Adhered to IMCI guide?		Univariable analysis		Multivariate analysis	
	No (*n* = 209)	Yes (*n* = 23)	UOR (95% CI)	*P* value	AOR (95% CI)	*P* value
	Number (%)	Number (%)				
Age group (years)						
Less than or equal to 32	111 (86.1)	18 (14.0)	1		1	
More than 32	98 (95.2)	5 (4.8)	0.31 (0.11–0.88)	0.027	0.63 (0.19–2.15)	0.463
Academic qualifications						
Certificate	182 (95.8)	8 (4.2)	1		1	
Diploma	24 (64.9)	13 (35.1)	12.32 (4.63–32.77)	<0.001	6.97 (1.82–26.67)	**0.005**
Degree	3 (60.0)	2 (40.0)	15.17 (2.21–103.89)	0.006	1.02 (0.10–10.43)	0.986
Time taken to follow the IMCI steps						
Long	96 (98.0)	2 (2.0)	1		1	
Short	113 (84.3)	21 (15.7)	8.92 (2.04–39.02)	0.004	12 (2.73–61.66)	**<0.001**
IMCI support supervision						
Never received	165 (92.2)	14 (7.8)	1		1	
Ever received	44 (83.0)	9 (17.0)	2.41 (0.98–5.94)	0.056	0.94 (0.27–3.24)	0.921
Inadequate staffing						
Yes	102 (96.2)	4 (3.8)	1		1	
No	107 (84.9)	19 (15.1)	4.53 (1.49–13.76)	0.008	2.96 (0.85–10.32)	0.089
Recent shortage of IMCI drugs at health facility						
Yes	158 (93.5)	11 (6.5)	1		1	
No	51 (81.0)	12 (19.0)	3.38 (1.41–8.12)	0.006	7.11 (2.37–21.38)	**<0.001**
The IMCI guideline is difficult to use						
Yes	100 (98.0)	2 (2.0)	1		1	
No	109 (83.9)	21 (16.1)	9.63 (2.20–42.13)	0.003	27.7 (5.40–142.25)	**<0.001**

*Note*. Percentages were calculated as *n*/*N* for each row; level of statistical significance was set less than 5%; AOR: adjusted odds ratio; UOR: unadjusted odds ratio.

## Abbreviations

AEC: Aweil East County
AIDS: Acquired immunodeficiency syndrome
AOR: Adjusted odds ratio
CHWs: Community Health Workers
CPE: Continuous Professional Education
DIB: Difficulty in breathing
HIV: Human Immunodeficiency Virus
HRH: Human Resources for Health
IMCI: Integrated Management of Childhood Illnesses
IQR: Interquartile range
NBEGS: Northern Bar El Ghazal State
PHCC: Primary health care center
PHCU: Primary health care unit
UOR: Unadjusted odds ratio
VIF: Variance inflation factor
WHO: World Health Organization.

## Additional Points

*Availability of Data and Materials. *The data set used in this study shall be provided on request in order to protect respondent anonymity.
